# Contextual factors in value-based decision support to enhance health technologies adoption: the case of biosimilars

**DOI:** 10.3389/fphar.2025.1599013

**Published:** 2025-08-13

**Authors:** Maximilian Otte, Zoltán Kaló, Hussain Abdulrahman Al-Omar, Meindert Boysen, Yingyao Chen, Ana Paula Beck Da Silva Etges, Guvenc Kockaya, Iñaki Gutierrez-Ibarluzea, Ahmed Mohammed Seyam, Deirdre Mullen, Hans-Peter Dauben

**Affiliations:** ^1^ OITBpathway V. o. G., Eupen, Belgium; ^2^ Institut für Gesundheit und Gesellschaft (I2GG), Cologne, Germany; ^3^ dkHealth, Cologne, Germany; ^4^ Center for Health Technology Assessment, Semmelweis University, Budapest, Hungary; ^5^ Center for Pharmacology and Drug Research & Development, Semmelweis University, Budapest, Hungary; ^6^ Syreon Research Institute, Budapest, Hungary; ^7^ Department of Clinical Pharmacy, College of Pharmacy, King Saud University, Riyadh, Saudi Arabia; ^8^ Health Technology Assessment International, Edmonton, AB, Canada; ^9^ School of Public Health, Fudan University, Shanghai, China; ^10^ Institute for Health Technology Assessment, Federal University of Rio Grande do Sul, Porto Alegre, Brazil; ^11^ Avant-garde Health, Boston, MA, United States; ^12^ Econix Research, Tallinn, Estonia; ^13^ Health ClusterNET, Amsterdam, Netherlands; ^14^ Universal Health Insurance Authority, Kairo, Egypt

**Keywords:** value-based decision making, multi-criteria decision analysis, health technology assessment, implementation, biosimilars

## Abstract

**Introduction:**

Biosimilar medicines play a critical role in enhancing global health outcomes by improving access to effective biologic treatments. However, their acceptance and implementation, particularly in emerging markets, depend not only on clinical evidence but also on the integration of societal, individual, and cultural values. This paper explores how value-based decision-making can support the adoption of biosimilars across diverse contexts.

**Methods:**

A multi-stakeholder workshop was conducted with participants from various countries, focusing on decision-making processes for biosimilars in emerging health systems. Discussions addressed stakeholder roles, contextual influences, and the alignment of evidence with values. A Multi-Criteria Decision Analysis (MCDA) framework was proposed as a tool to systematically integrate measurable outcomes and intangible factors such as trust, perceived quality, and cultural acceptance.

**Results:**

Key barriers identified included regulatory uncertainties, limited local evidence, regional data protection constraints, and patient preferences for originator biologics. Participants emphasized the importance of adaptable frameworks that reflect local cultural, economic, and systemic conditions. The proposed MCDA approach was viewed as a promising method for capturing complex value dimensions and facilitating transparent, inclusive decision-making. Broader societal benefits of biosimilars, such as economic development through local production, were also highlighted.

**Discussion:**

The workshop underscored the need for value-sensitive implementation strategies that go beyond clinical effectiveness. Integrating context-specific values into evidence-based decision-making can foster trust and support the sustainable adoption of biosimilars. The MCDA framework offers a structured approach to operationalize these principles. Future research should test and refine this model in varied health system settings to support its practical application by policymakers, healthcare providers, and industry stakeholders.

## Introduction

Health technologies play an important role in enhancing global health outcomes by presenting novel opportunities for intervention and prevention. However, their acceptance and implementation are contingent upon not only study-based evidence and evidence-based decision support, as employed in Health Technology Assessment (HTA), but also the consideration of a diverse range of individual, societal, and cultural values and preferences (Damschroder et al., 2022). These values, shaped by cultural, societal, and personal attitudes, experiences, and beliefs, exert influence at various levels and constitute a fundamental basis for the successful implementation and acceptance of health technologies (Grossi et al., 2021; Trowman et al., 2023; AlQudah et al., 2021). They create an individual and unique contextual environment (Metallo et al., 2022; Mahlich et al., 2018; Alagöz et al., 2011).

While HTA systems primarily rely on systematic and study-based criteria to support decisions, such as the justification of technology reimbursement, individual, societal, and cultural values may not be fully and unanimously captured in a single dimension in different jurisdictions (Stich et al., 2019). The VALIDATE-HTA approach has underscored the significance of these values in decision-making processes (Oortwijn and Sampietro-Colom, 2022). These values are crucial for the acceptance of novel technologies in clinical or healthcare settings (Nittas et al., 2024). A more comprehensive understanding of these context-specific values could not only enhance the argumentation for certain technologies but also bridge the gap between the value proposition of a technology and its actual perception, acceptance, and implementation.

Emphasising the incorporation of values into decision-making processes, practical implementation reveals persistent challenges in the acceptance and adoption of technologies in clinical and healthcare systems (European Comission, 2022; O'Rourke et al., 2020). These challenges underscore the necessity of fostering a more sophisticated dialogue on how policymakers can integrate study-based evidence and a nuanced comprehension of context-sensitive values. Addressing these complexities necessitates a systematic examination of decision-making processes and stakeholder roles, as well as the exploration of tools and methodologies to bridge the gap between theoretical frameworks and practical implementation (Strifler et al., 2020; McNett et al., 2024).

The question which are taken into consideration are

• Which values are important in decision-making processes?
• Which stakeholders play a role and what positions do they take?
• Can a common framework of values be defined that can be applied in different contexts?
• Does such a framework require a context-specific weighting?
• Can Multi-Criteria Decision Analysis (MCDA) as a method to collect evidence beyond studies including practical approaches and integration of context related topics and values
• Which tools are suitable for validating such a framework?

These questions had been the discussed within an international workshop on value-based decision making for biosimilars and its inclusion in a wide range of markets. This paper consolidates the contributions from this workshop.

## Methodology

A full day workshop aimed to achieve consensus on critical issues, supported by a pre-reading phase to enhance participants’ awareness of the key questions and provide relevant contextual insights, for cross-continent markets.

### Use case selection

Biosimilars were selected as the focus due to their demonstrated ability to improve patients’ outcomes, reduce healthcare costs and improve treatment access particularly in low- and middle-income countries (LMIC) (European Medicines Agency, 2023; Mulcahy et al., 2018; Otte et al., 2024; Almutairi et al., 2023; Alnaqbi et al., 2024). Despite strong evidence supporting their economic and clinical benefits, significant global disparities in adoption persist (Cazap et al., 2018; Chang et al., 2023; Cohen et al., 2016; Gasteiger et al., 2021; Jacobs et al., 2016; McCamish and Woollett, 2011; Moorkens et al., 2017; Weise et al., 2012; Yang et al., 2022). These variations highlight the need to examine contextual factors influencing decision-making processes.

Participants were encouraged to share their experiences and reflect on regional practices, recognising that not all relevant information is explicitly available in the literature. To facilitate discussions, case studies were provided, examining the interplay of economic, clinical, regulatory and other stakeholder values in biosimilar adoption.

### Scientific background through literature review and presentations

A narrative literature review provided a foundation for the workshop, focusing on two key topics:

1. Values in Decision-Making Processes: Understanding how societal and individual values influence health technology implementation and acceptance.
2. Biosimilars: Examining global adoption trends, barriers, facilitators, and their clinical, economic, and societal value.

The review, conducted through a critical appraisal process, synthesized findings to support the biosimilars use case. These results were shared with participants in a preparatory document, helping contextualise discussions and address scientific questions.

### Expert selection for presentation and participation

To ensure a multidisciplinary and globally representative dialogue, experts were selected based on:

• Expertise in HTA and Health Economics
• Practical involvement in decision-making, clinical implementation, or procurement and reimbursement processes
• At least 5 years of experience in health technology-related decisions

A diverse group of participants took part in this workshop including researchers, clinicians, health authority representatives, and procurement specialists. Global diversity was emphasised, incorporating experts from Africa, Asia and Oceania, Europe as well as Latin America, ensuring perspectives from countries with varying populations, Gross Domestic Product (GDP) levels and cultural contexts.

### Multi-criteria collection tools

To capture and integrate diverse contextual values, MCDA was proposed as an appropriate methodology. Widely used in HTA, MCDA addresses complex decision-making problems by integrating quantitative and qualitative dimensions within a unified framework (Frazão et al., 2018; Marsh et al., 2017; Oliveira et al., 2019; Jakab et al., 2020; Kolasa et al., 2016a; Németh et al., 2019; Inotai et al., 2018; Kolasa et al., 2016b; Elezbawy et al., 2022; Farghaly et al., 2021). While MCDA is sometimes criticised as “too complex or mechanistic,” (van Til et al., 2014) it was considered essential for this initiative due to its ability to balance measurable outcomes with intangible factors like societal and cultural values (Vásquez et al., 2024).

#### Workshop

The workshop, titled “Value-based Decision-Making: Biosimilars and Off-Patent Medicines in Health Services,” was held on 16 November 2024, in Barcelona, Spain. Following a structured methodology, the workshop incorporated pre-workshop literature-based preparation, expert presentations in the first part, and interactive discussions in the second part.

The workshop followed a semi-structured, participatory format designed to encourage open and balanced contributions while minimising bias. Each session was moderated by an independent facilitator with methodological expertise in decision-supportive actions and biosimilars. These facilitators supported equitable speaking time, moderated divergent opinions and clarified misunderstandings.

To guide the discussions, standardised topic guides were used, which were informed by the pre-circulated pre-reading document.

Following each roundtable session, a structured consensus-building process was employed using verbal confirmation of agreed themes. Disagreements were documented and presented during the plenary for transparent discussion using a virtual whiteboard approach. To further mitigate potential bias, no representatives from the workshop sponsoring organisation participated in the discussion or facilitation. The final synthesis of results was conducted by the academic organisers based on verbatim notes and written participant feedback forms collected at the end of the event.

During the first part of the workshop, participants explored values critical to decision-making processes and their contextual weighting in different healthcare systems. Presentations were followed by topic-specific roundtable discussions, enabling deeper exploration of key issues. The workshop concluded with an evaluation of participants’ perceptions, ensuring feedback could inform future initiatives.

#### Common opinion formulation

The second part of the workshop focused on identifying common themes, their relative importance, and strategies for integrating them into a global framework. This framework was designed to reflect diverse contextual weighting of values while maintaining robust scientific alignment.

#### Key themes included

• Values in Decision-Making Processes: Examining how societal and individual values influence the adoption of biosimilars.
• Procurement Processes: Analysing procurement practices and their alignment with value-based principles.
• Decision Management: Evaluating frameworks for integrating diverse values, enhancing transparency, and achieving stakeholder acceptance.

Roundtable discussions were enriched by additional country experts, who provided regional insights, ensuring that the outcomes were globally relevant and context sensitive.

## Results

The workshop provided insights into the factors influencing the implementation of biosimilars, broadening the evidence-based perspective by emphasising contextual values essential for their integration in healthcare systems. The discussions addressed aspects, including the values underpinning decision-making, the roles of stakeholders, the feasibility of a common framework, the need for context-specific weighting, and the validation of such a framework using appropriate tools.

Participants identified trust as a fundamental value in decision-making processes. It was noted that trust could be strengthened through measures such as local production and the presence of well-established manufacturers. However, concerns about quality and safety emerged as key barriers, particularly regarding extrapolated indications and regulatory uncertainties—despite the lack of supporting evidence from clinical studies or real-world data (Marsh et al., 2017). Cultural acceptance was also highlighted as an important factor, as preferences for original biologics persist even in countries with a strong biosimilar manufacturing sector. To address these cultural challenges, participants suggested educational initiatives and the integration of biosimilars into clinical practice guidelines.

Participants emphasised the important role of scientists and clinical experts in the biosimilars decision-making process. They provide evidence on biosimilars’ safety and efficacy, which forms the basis for regulatory decisions. Clinical experts are particularly important in extrapolating indications for biosimilars, especially during adoption, when specialised knowledge and judgment are required.

Participants reported about regional preferences for local safety and efficacy data and international data may not always be accepted by regulators or clinicians. This was about scepticism about the transferability of international regulatory data to a respective local context. To address this, participants proposed identifying regions with higher scepticism early in the biosimilar development process. This would allow for targeted clinical evaluations in those regions while transferring data to regions with lower levels of scepticism, thereby reducing additional workload and contributing early to adoption barriers.

Procurement was highlighted as having a role in implementation and adoption, as it often involves criteria beyond the healthcare sector. Participants reported that procurement decisions increasingly consider factors such as strengthening local supply chains, supporting local suppliers and manufacturers and promoting local economic development. In this regard, participants emphasised the added value biosimilars could provide in other policy areas, such as promoting economic growth through local production, creating jobs, and fostering regional innovation. The Health for all Policies concept was mentioned in this regard supporting these considerations of co-benefits between health and other policies (Greer et al., 2022).

At the same time, participants noted that patients without financial disincentives through higher copayments show a strong preference for originator biologics, which can limit the adoption of biosimilars. One participant describes out of his context, that such resistance can persist even when biosimilars are produced domestically and therefore could be perceived as a somewhat local and normal product.

The discussions also identified operational barriers in implementation and adoption, such as the need for disinvestment processes to manage the simultaneous presence of biosimilars and originators in the market, were highlighted (Alnaqbi et al., 2024; Fasseeh et al., 2023; Inotai et al., 2017). One participant mentioned that in emerging markets disinvestment activities such in Malaysia could contribute to overcome operational barriers (Kamaruzaman et al., 2024). Ensuring a stable supply chain was seen critical to building trust and acceptance.

Participants discussed the development of a common framework to integrate the mentioned values into health services. They emphasised that such a framework must be adaptable to different cultural, social, and economic contexts. Flexibility was highlighted as essential, allowing the framework to address systemic goals while accommodating individual needs through a combination of top-down and bottom-up approaches. Pragmatism was also emphasised, with a focus on creating user-friendly processes that enable actionable decisions and the implementation of concrete measures.

To disclose and validate the contextual differences and relative importance of potentially relevant value criteria, MCDA was identified as a suitable tool by the participants. A common opinion could be reached that the systematically recording and weighting of values with the involvement of all stakeholder groups will contribute to make decision-making processes more transparent and comprehensible. The method was recognised as particularly valuable for integrating practical approaches and context-specific topics into local decision frameworks, addressing the complexities of biosimilar adoption.

## Discussion

This paper explored the integration of values into decision-making processes for health technologies, using biosimilars as a case study. This continues a discussion initiated during an international workshop held in Copenhagen in 2023 (Otte et al., 2024). The workshop provided insights into how societal, individual, and cultural values, alongside evidence-based approaches, influence the acceptance and implementation of health technologies. As raised already by other authors, the findings underscored the complexity of decision-making, revealing the central role of values such as trust, quality, safety, and cultural acceptance, which significantly affect stakeholder perceptions and actions (Trowman et al., 2023; Metallo et al., 2022; Mahlich et al., 2018; Oortwijn and Sampietro-Colom, 2022).

This study draws on the outcomes of a single-day international workshop involving a limited but diverse group of expert participants. While this diversity enriched the discussion, the sample size limits the generalisability of the findings across all emerging markets. The findings should therefore be interpreted as indicative rather than representative. Furthermore, participant selection may have introduced an element of expert bias toward stakeholders with a particular interest in health technology assessment and value-based approaches. Nevertheless, the study provides a tested conceptual and methodological foundation for further workshops for integration of context-sensitive values in a structured discussion process. While insights from diverse regions were incorporated, the systematic identification and analysis of specific regional nuances will be the focus of a follow-up workshop.

In terms of the research questions raised, key stakeholders identified in the process—including healthcare practitioners, researchers, regulators, procurement experts, and patients—play diverse and interconnected roles. Their contributions range from providing evidence-based insights to shaping The workshop demonstrated that creating a common framework for value-based decision support is feasible but must accommodate cultural, economic, and societal differences through context-sensitive weighting and flexible approaches.

MCDA was as an effective tool for systematically capturing and integrating multiple values. As already described by Inotai et al. its ability to combine both objective evidence and subjective considerations enhances transparency and decision-making accuracy (Inotai et al., 2018). However, MCDA’s success depends on clear problem definition and the alignment of tools with specific decision-making objectives (Marsh and Bhashyam, 2015).

The findings aligned with existing literature, emphasising the importance of context-sensitive approaches in implementing health technologies. The inclusion of sociocultural values bridges the gap between clinical evidence and societal acceptance, addressing barriers such as regulatory challenges, scepticism, and systemic resistance to change. Furthermore, the workshop highlighted broader societal benefits of biosimilars, including economic growth through local production, job creation, and regional innovation (Napier et al., 2017).

Despite the workshop’s limited participant pool, which allowed for in-depth discussion but not exhaustive representation, the results offer a foundation for future research. Context-specific studies should further validate these findings, incorporating profiling of local circumstances and values as part of global efforts supported by networks and public agencies.

These conclusions have significant implications for policymakers, regulators, and healthcare providers, as well as innovators and industry stakeholders. By recognising the importance of values, decision-makers can develop inclusive adoption strategies that address both evidence and context, ultimately improving the implementation of biosimilars and other health technologies. A combined bottom-up and top-down approach—“understanding from below, acting from above”—is recommended to ensure both grassroots and systemic alignment (Inotai and Kaló, 2019).

Future research efforts should focus on the development of practical methods to define ‘context profiles and context-specific reasoning chains that combine formal evidence requirements with societal and individual values. The creation of these context profiles is an essential complement to early detection measures as described in the EuroScan International Network Toolkit (Assembly, 2015). In particular, the forthcoming version of the toolkit, to be published in 2025, emphasises the need to understand the local context early and comprehensively - long before health technologies are introduced. This allows a balance to be struck between quantitative and qualitative insights, with tools such as MCDA acting as a bridge between these perspectives.

The aim is to translate complex science that is still poorly understood into formats that are understandable and actionable for decision-makers and other stakeholders who are not methodological experts. This promotes evidence-based decision-making that goes beyond formal evidence criteria while taking context-specific factors into account.

Evidence is global, but decisions are made locally. A structured framework, such as the one proposed here, will be particularly useful for introducing health technologies like biosimilars, where evidence and adoption rates may vary by region.

No one-size-fits-all approach exists; instead, national health priorities—be they economic or clinical—must guide local implementation strategies. Interactive workshops formats and the involvement of key stakeholders as described in this paper will be essential for identifying contextual factors early and merging scientific accuracy with practical applicability. Over time, this will contribute to more equitable, effective, and sustainable healthcare systems globally (Assembly, 2015).

## Conclusion

The findings of this study highlight the role of context in decision-making processes for health technologies. While clinical and economic evidence provides an foundation, factors such as trust, cultural acceptance, regulatory structures and local production capacities can influence the adoption of biosimilars. The workshop demonstrated that Multi-Criteria Decision Analysis (MCDA) offers a promising and transparent approach to integrate both quantitative data and qualitative values, such as stakeholder preferences and societal priorities, into structured decision frameworks.

A key outcome was the recognition that although a globally applicable value-based framework is feasible, it must remain adaptable to regional specificities. Context-sensitive weighting and stakeholder-specific modifications are important to address the unique barriers and priorities of different countries. Broad and inclusive stakeholder engagement including clinicians, regulators, procurement specialists and patients emerged as a prerequisite for effective and legitimate implementation strategies, especially in culturally diverse or resource-constrained settings.

Beyond clinical benefits, biosimilars were acknowledged for their potential to contribute to broader societal goals, including local economic development, job creation, and innovation. However, successful adoption requires early recognition and management of operational challenges such as supply chain stability, disinvestment processes, and scepticism toward externally generated data.

Future research should concentrate on the development of context profiles and context-specific reasoning chains. These tools are necessary to bridge the gap between formal evidence requirements and real-world implementation, thereby improving the global transferability and adoption of health technologies in a contextually appropriate manner.

## Data Availability

The raw data supporting the conclusions of this article will be made available by the authors, without undue reservation.
